# Outcomes after robot-assisted radical cystectomy with orthotopic neobladder in women

**DOI:** 10.1007/s00345-024-05339-w

**Published:** 2024-11-02

**Authors:** Juhana Rautiola, Alberto Martini, Laura S Mertens, Viktor Skokic, Luca Di Gianfrancesco, Carlo Andrea Bravi, Julia Heinzelbecker, Mikolaj Mendrek, Stephan Buse, Guillaume Ploussard, Hubert John, Abdullah Erdem Canda, Mevlana Derya Balbay, Sebastian Edeling, Charles Van Praet, Sami-Ramzi Leyh-Bannurah, Alexander Mottrie, Frederiek D’Hondt, Hendrik van der Poel, Camille Berquin, Karel Dacaestecker, Richard Gaston, Peter Wiklund, Abolfazl Hosseini

**Affiliations:** 1 Department of Molecular Medicine and Surgery, Department of Pelvic Cancer, Karolinska Institutet, Karolinska University Hospital, Stockholm, SE-171 76 Sweden; 2 Department of Urology, University of Cincinnati, Cincinnati, OH USA; 3 Department of Urology, Netherlands Cancer Institute, Amsterdam, The Netherlands; 4 Oncological Urology, IRCCS Veneto Institute of Oncology, Padua, Italy; 5 Department of Urology, The Royal Marsden NHS Foundation Trust, London, UK; 6 Department of Urology, OLV Hospital, Aalst, Belgium; 7 Orsi Academy, Ghent, Belgium; 8 Department of Urology and Pediatric Urology, Saarland University Medical Center and Saarland University, Homburg/Saar, Germany; 9 Department of Urology, Urologic Oncology and Robot-assisted Surgery, St. Antonius Hospital, Gronau, Germany; 10 Department of Urology, Alfried Krupp Krankenhaus, Essen, Germany; 11 Department of Urology, La Croix du Sud Hospital, Toulouse, France; 12 Department of Urology, Institut Universitaire du Cancer Toulouse-Oncopôle, Toulouse, France; 13 Department of Urology, Cantonal Hospital Winterthur, Winterthur, Switzerland; 14 Department of Urology, Koç University School of Medicine, Istanbul, Turkey; 15 Department of Urology, Vinzenz Hospital, Hannover, Germany; 16 Department of Urology, Ghent University Hospital, Ghent, Belgium; 17 Department of Urology, Amsterdam University Medical Centers, Amsterdam, The Netherlands; 18 Department of Urology, AZ Maria Middelares Hospital, Ghent, Belgium; 19 Department of Urology, Clinique Saint Augustin, Bordeaux, France; 20 Department of Urology, Icahn School of Medicine at Mount Sinai, New York, USA; 21 Department of Molecular Medicine and Surgery, Karolinska Institutet, Stockholm, Sweden; 22 Department of Urology, Danderyds Hospital, Stockholm, Sweden; 23 Department of Urology, Basel University Hospital, Basel, Switzerland

**Keywords:** Female cystectomy, Functional outcomes, Pelvic organ-preserving cystectomy, Orthotopic neobladder, Robot-assisted radical cystectomy

## Abstract

**Purpose:**

To investigate functional, oncological and complication outcomes in women undergoing robot-assisted cystectomy (RARC) with intracorporeal orthotopic neobladder.

**Methods:**

From a multi-institutional database, we identified females with bladder cancer treated with RARC and intracorporeal orthotopic neobladder. We evaluated the continence rate, short-term oncological outcomes, and complication rates. Analyses were repeated and stratified by the status of preserving gynecological organs.

**Results:**

The study involved 146 patients with the median age 60 years (IQR, 51–66 years). Pelvic organ-preserving procedure (POP) was performed in 77 patients (53%). Overall daytime and nighttime continence rates were 54% and 53%, respectively. For POP, the continence rate was 58% for both daytime and nighttime continence. In the non-POP cohort, the continence rate was 50% for daytime and 49% for nighttime continence. Both groups had balanced positive surgical margin rates (5,3% for POP and 4,7% for non-POP). In the whole cohort, high-grade (Clavien-Dindo ≥3) early and late complication rate was 7,5% and 7,5%, respectively.

**Conclusions:**

Robot-assisted radical cystectomy with intracorporeal orthotopic neobladder in females demonstrate excellent functional and complication outcomes. Pelvic organ-preserving cystectomy enhances urinary continence rates without adversely affecting surgical margins. Orthotopic neobladder in selected women with bladder cancer, along with pelvic organ-preserving cystectomy may be used for improved functional outcomes without compromising oncological results.

**Supplementary Information:**

The online version contains supplementary material available at 10.1007/s00345-024-05339-w.

## Introduction

Radical cystectomy (RC) with urinary diversion remains the standard-of-care for muscle-invasive bladder cancer (MIBC) and for selected patients with high-risk non-MIBC [1]. Women are three to four times less likely to develop bladder cancer than men [2], but are often diagnosed with more advanced disease [3]. Traditionally, anterior pelvic exenteration, which includes the removal of the anterior wall of the vagina, hysterectomy and removal of fallopian tubes and ovaries, has been performed in addition to cystectomy. The concern with preserving pelvic organs is mainly contributed to oncological results, fearing preoperative understaging, positive surgical margins and an elevated risk of local recurrencies. However, advancements in preoperative assessment, the adoption of minimally invasive surgical techniques and enhanced comprehension of pelvic anatomy with the increased awareness of quality-of-life issues have led to the introduction of a more pelvic organ-preserving approach.

The two most common urinary diversions are the ileal conduit and the orthotopic neobladder. Advantages of ileal conduit are shorter operative time and less complicated surgical procedure. Yet, many studies demonstrate better quality-of-life with comparable mortality and morbidity rates after orthotopic neobladder reconstruction [4–7]. Historically, however, neobladder is less frequently performed in women than in men [8]. Functional impairments, such as sexual and urinary dysfunction are common after RC in both sexes, although most studies have primarily focused on post-prostatectomy outcomes in men [9]. There is limited research on functional outcomes in women after cystectomy. Indeed, pelvic organ-preserving procedure (POP) at the time of RC and orthotopic neobladder demonstrates comparable oncological outcomes with non-POP with good functional outcomes in a selected population [10, 11]. The limitation of the articles reviewed by this systematic review was the small sample size of the studies with relatively short follow-up [11].

The aim of this study is to provide a more comprehensive understanding of functional, oncological, and complication outcomes following RARC and intracorporeal orthotopic neobladder reconstruction in females with bladder cancer. This study seeks to address the current gap, and, to the best of our knowledge, stands as the most extensive study to date investigating these outcomes.

## Materials and methods

### Study population

This retrospective study included female patients with bladder cancer (BCa) who underwent RARC with intracorporeal orthotopic neobladder reconstruction. Data was collected from a multi-institutional database comprising tertiary referral centers in Europe and in the U.S. We included patients with available data and at least 12 months follow-up time after the surgery. The study was sponsored by the EAU Robotic Urology Section (ERUS) Scientific Working Group.

Additionally, information on pelvic organ-preserving procedure (POP) was gathered to determine if POP improves functional outcomes without affecting oncological outcomes. Pelvic organ-preserving procedure was defined as the preserving of at least the vagina and the uterus during cystectomy. The patients were categorized into two groups based on the POP. Daytime and nighttime urinary continence were recorded separately. Urinary continence was defined as the patient requiring maximum one daily pad. The use of pads or condom devices was recorded. Data on oncological outcomes were reported for the entire cohort and stratified by POP. Complications were divided to early (1–29 days) and late (30–90 days) postoperative complications according to Clavien-Dindo classification, where the most severe complication grade was recorded for an individual patient. In addition, early (1–29 days) and late (30–90) postoperative readmission rates were collected. Data variables were collected from electronical medical records (EMR).

### Statistical analysis

All analyses were conducted for the entire cohort and stratified by POP or non-POP. Median values with interquartile range (IQR) were reported for continuous variables, while categorical variables were presented as percentages. To assess the factors influencing urinary continence rates, a multivariable logistic regression was conducted to predict urinary continence at 12 months after surgery. Here, also patients with hypercontinence were counted in. To assess differences between POP and non-POP groups, the Chi-square test and Fisher’s exact test were used for categorical variables, and the Mann-Whitney test was utilized for continuous variables. The likelihood ratio test was used to calculate p values in the multivariate models. A *P*-value < 0,05 was considered statistically significant. All tests were performed in R version 4.0.0. Our study was approved by the Regional Ethics Committee in Stockholm (DNR: 2016/1836-31/1). Informed consents were not collected, and the data used for analyses were pseudonymized.

## Results

A total of 146 female patients with bladder cancer who underwent RARC and intracorporeal orthotopic neobladder reconstruction were included in the study. The median age of the patients was 60 years (IQR, 51–66 years), the median operative time was 330 min (IQR, 288–399 min) and the median length of stay was 9 days (IQR, 7–14 days). Pelvic organ-preserving procedure was performed in 77 patients (53%) and was statistically associated with shorter length of stay as compared to non-POP (8 days vs. 14 days, *P* < 0,001). All urinary diversions were performed intracorporeally, with 73% of patients undergoing a Studer/Wiklund neobladder reconstruction technique, and all patients in the POP cohort had Studer/Wiklund as the reconstruction technique. Patient characteristics are presented in Table 1.

**Table 1:** Patient characteristics

	All (*n* = 146)	Organ-preserving (*n* = 77, 55%)	Non-organ-preserving (*n* = 64, 45%)	*P*-value
Age at operation, median - (IQR)	61 (54–67)	59 (50–66)	62 (56–67)	0,03
ASA score, n (%)				0,33
• 1	33 (22)	14 (18)	18 (28)	
• 2	84 (58)	48 (62)	33 (52)	
• 3	29 (20)	15 (20)	13 (20)	
Median BMI, kg/m^2,^ median – (IQR)	23 (21–26)	23 (21–26)	23 (20–26)	0,41
cT stage, n (%)				0,001
• a/1/CIS	56 (38)	38 (49)	15 (23)	
• 2	73 (50)	28 (36)	43 (67)	
• 3	16 (11)	10 (13)	6 (9)	
• 4	1 (1)	1 (1)	0 (0)	
MIBC/NMIBC				0,03
• MIBC	90 (62)	39 (51)	49 (77)	
• NMIBC	56 (38)	38 (49)	15 (23)	
NAC • Yes • No	71 (49) 75 (51)	36 (47) 41 (53)	34 (53) 30 (47)	0,56
ypT stage (%)				0,20
• ypT0	31 (44)	15 (42)	16 (47)	
• ypTa/1/CIS	9 (13)	7 (19)	2 (6)	
• ypT2	15 (21)	8 (22)	6 (18)	
• ypT3	15 (21)	5 (14)	10 (29)	
• ypT4	1 (1)	1 (3)	0	
pT stage, n (%)				0,47
• pT0	22 (30)	14 (36)	8 (27)	
• pTa/1/CIS	30 (41)	17 (44)	10 (33)	
• pT2	8 (11)	4 (10)	4 (13)	
• pT3	8 (11)	3 (8)	5 (17)	
• pT4	5 (7)	1 (3)	3 (10)	
PLND template, n (%) • No	3 (2)	2 (3)	0 (0)	0,05
• Limited	5 (4)	5 (7)	0 (0)	
• Standard	29 (22)	18 (25)	10 (18)	
• Extended	93 (71)	45 (63)	46 (82)	
• Super-extended • Missing	1 (1) 15	1 (2) 6	0 (0) 8	
Lymph node status, n (%)				0,24
• Negative	123 (86)	68 (90)	51 (81)	
• Positive • Missing	20 (14) 3	8 (10) 1	12 (19) 1	
Soft tissue margins, n (%)				1,00
• Negative	137 (95)	71 (95)	61 (95)	
• Positive • Missing	7 (5) 2	4 (5) 2	3 (5) 0 (0)	
Neobladder technique • Studer/Wiklund • Hautmann • Gaston	102 (73) 7 (5) 23 (16)	72 (100) 0 (0) 0 (0)	26 (41) 7 (11) 23 (36)	-
Operative time, median - (IQR)	330 – (288–399)	360 – (300–420)	300 – (270–370)	0,007
Perioperative bleeding, median -(IQR)	150 – (90–300)	150 – (50–250)	200 – (100–300)	0,05
Length of stay, median - (IQR)	9 (7–14)	8 (7–10)	14 (9–18)	> 0,001

### Urinary continence

In the whole cohort, a total of 51 patients (38%) were pad free, eight patients (6%) needed security pad, 14 patients (10%) reported the use of one daily pad, and 30 patients (22%) were hypercontinent. If these groups were considered together, the daytime continence rate was 76%. In the organ-preserving cohort, 29 patients (41%) were pad free, four patients (6%) used security pad, eight patients (11%) used one daily pad, and 20 patients (28%) were hypercontinent. Again, if these groups were considered together, the continence rate was 86%. In the non-organ-preserving cohort, 21 patients (35%) were pad free, three patients (5%) used security pad, six patients (10%) used one pad per day, and nine patients (15%) were hypercontinent. If considered together, the daytime continence rate was 65%. Furthermore, 33 patients (24%) in the whole cohort, 10 patients (15%) in the POP cohort and 23 patients (35%) in the non-POP cohort reported the use of more than two daily pads.

Regarding nighttime continence, in the whole cohort, 41 patients (31%) were pad free, seven patients (5%) needed security pad, 22 (17%) reported the use of one pad overnight, and 24 patients (18%) were hypercontinent. If these groups were considered together, the nighttime continence rate was 71%. In the POP cohort, 24 patients (34%) were pad free, four patients (6%) used security pad, 13 patients (18%) used one pad overnight, and 18 patients (25%) were hypercontinent. If considered together, the continence rate was 83%. In the non-POP cohort, 16 patients (28%) were pad free, three patients (5%) used a security pad, nine patients (16%) used one pad per night, and five patients (9%) were hypercontinent. If considered together, the nighttime continence rate was 58%. Furthermore, 39 patients (30%) in the whole cohort, 12 patients (17%) in the POP cohort and 25 patients (43%) in the non-POP cohort reported the use of uridom or two or more pads overnight. In multivariable analysis, the pelvic organ-preserving procedure was associated with better day- and nighttime continence (OR 3,6; 95%CI 1,4–10,2 for daytime and OR 3,5; 95% CI 1,4–8,8 for nighttime continence, respectively). Tables 2 and 3 demonstrate detailed information on urinary continence rates.

**Table 2:** The rate of urinary continence at one year after surgery

	All, *n* (%)	POP, *n* (%)	Non-POP, *n* (%)	*P*-value
Daytime continence				
• Pad free	51 (38)	29 (41)	21 (35)	
• Security pad	8 (6)	4 (6)	3 (5)	
• 1 pad	14 (10)	8 (11)	6 (10)	
• 2 daily pads	11 (8)	6 (9)	4 (7)	0,015
• ≥ 3 daily pads	21 (16)	4 (6)	17 (28)	
• Hypercontinent • Missing	30 (22) 11 (8)	20 (28) 6 (8)	9 (15) 4 (6)	
Nighttime continence				
• Pad free	41 (31)	24 (34)	16 (28)	
• Security pad	7 (5)	4 (6)	3 (5)	
• 1 pad	22 (17)	13 (18)	9 (16)	
• 2 pads	16 (12)	5 (7)	10 (17)	
• ≥ 3 pads	22 (17)	6 (9)	15 (26)	0,013
• Uridom	1 (1)	1 (1)	0 (0)	
• Hypercontinent • Missing	24 (18) 13 (9)	18 (25) 6 (8)	5 (9) 6 (9)	

**Table 3:** Multivariable analysis predicting urinary continence

Variable			Multivariable				
	Daytime				Nighttime		
	OR	95% CI		*P*-value	OR	95% CI	*P*-value
**Age** • Age under 50 (reference)	-				-		
• Age group 50–60	1,5	0,3–8,4 0,3–8,5 0,2–10,3			1,5	0,4–6,7 0,3–5,3 0,2–7,8	
• Age group 60–70	1,5				1,1		
• Age group over 70	1,4			0,96	1,2		0,92
**ASA class** • ASA class 1 (reference)	-				-		
• ASA class 2	0,6	0,2–2,0 0,2–3,1			0,9	0,3 − 2,6 0,3–3,8	
• ASA class 3	0,7			0,74	1,0		0,95
**BMI**							
• BMI under 20	0,8	0,2–3,3			1,0	0,3–3,6	
• BMI 20–30 (reference)	-	-			-	-	
• BMI 25–30	1,7	0,6 − 5,1			0,7	0,2–2,2	
• BMI 30–35	0,8	0,2–3,3		0,71	0,7	0,2–2,7	0,92
**cT status – ≤cT2 (reference)**							
• Locally advanced cancer (≥T3)	1,4	0,3–5,2		0,67	0,9	0,2–3,2	0.88
**Organ-preserving operation –yes (reference)**							
• Organ-sparing	3,6	1,4–10,2		0,007	3,5	1,4–8,8	0,006

### Oncological outcomes

In the pelvic organ-preserving cohort 38 patients (49%) had non-muscle-invasive bladder cancer as their clinical T-stage as compared to 15 patients (23%, *P* = 0,003) in the non-POP cohort (Table 1). Neoadjuvant chemotherapy was administered to 49% of the patients, and in the final pathology report 44% of patients in the whole cohort had ypT0. Correspondingly, 42% of the patients in POP cohort and 47% in the non-POP cohort had ypT0 (Table 1). Both groups had similar rates of positive surgical soft tissue margins and lymph node-positive disease (Table 4). Furthermore, most of the patients (98%) underwent lymph node dissection (LND), extended LND being the most frequent extent of procedure (93 patients, 71%) and performed more often in patients with non-POP (82% vs. 63%, *P* = 0,047).

**Table 4:** Complication rates after RARC with orthotopic neobladder reconstruction

Covariate	All *n* (%)	Organ-preserving *n* (%)	Non-organ-preserving *n* (%)	*P*-value
Classification of early complications				0,02
• CD 1	11 (7,5)	9 (11,7)	1 (1,6)	
• CD 2	42 (28,8)	21 (27,3)	21 (32,8)	
• CD 3a	4 (2,7)	0 (0)	4 (6,2)	
• CD 3b	6 (4,1)	2 (2,6)	4 (6,2)	
• CD 4a	1 (0,7)	1 (1,3)	0	
• CD 4b	0	0	0	
• CD 5	0	0	0	
• No complication	82 (56,2)	44 (57,1)	34 (53,1)	
Early readmissions • Yes • No • Missing	32 (23,0) 107 (77,0) 7 (4,8)	16 (21,9) 57 (78,1) 4 (5,2)	16 (25,4) 47 (74,6) 1 (1,6)	0,78
Early reoperation • Yes • No • Missing	5 (4,1) 118 (95,9) 23 (15,8)	0 (0) 76 (100) 1 (1,3)	4 (9,3) 39 (90,7) 21 (32,8)	0,02
Classification of late complications				0,50
• CD 1	4 (2,7)	3 (3,9)	0 (0)	
• CD 2	13 (8,9)	6 (7,8)	7 (10,9)	
• CD 3a	4 (2,7)	3 (3,9)	1 (1,6)	
• CD 3b	7 (4,8)	3 (3,9)	4 (6,2)	
• CD 4a	0	0	0	
• CD 4b	0	0	0	
• CD 5	0	0	0	
• No complication	118 (80,8)	62 (80,5)	52 (81,2)	
Late readmissions • Yes • No • Missing	24 (21,1) 90 (78,9) 32 (21,9)	8 (11,4) 62 (88,6) 7 (9,1)	16 (39) 25 (61) 23 (35,9)	0,002
Late reoperation • Yes • No • Missing	6 (5,0) 113 (95,0) 27 (18,5)	1 (1,4) 73 (98,6) 3 (3,9)	5 (12,2) 36 (87,8) 23 (35,9)	0,02

### Complications

In the whole cohort, eleven patients (7,5%) had high-grade (Clavien-Dindo ≥3) early complication. The most common early complication was urinary tract infection (30 patients, 21%). Correspondingly, three patients (3,9%) in the POP cohort and eight patients (12,4%) in the non-POP cohort experienced early high-grade complications. In the whole cohort, eleven patients (7,5%) had high-grade (Clavien-Dindo ≥3) late complications. Similarly, urinary tract infection was the most common late complication (11 patients, 7,5%). Furthermore, six patients (7,8%) in the POP cohort and five patients (7,8%) in the non-POP had high-grade (Clavien-Dindo ≥3) late complication (Table 4). Description of the complications grouped by category are shown in Table 5.

**Table 5:** Early and late high-grade complications grouped by category

Complication category	All	Organ-preserving	Non-organ-preserving	*P*-value
**Gastrointestinal**, **ileus**				
Early	2 (1,4)	0 (0,0)	2 (3,1)	0,20
Late	-	-	-	
**Gastrointestinal**,** internal herniation**				
Early	-	-	-	-
Late	1 (0,7)	1 (1,3)	0 (0,0)	1,00
**Genitourinary**,** ileoneobladder fistula**				
Early	1 (0,7)	0 (0,0)	1 (1,6)	0,45
Late	-	-	-	-
**Genitourinary**,** enterovaginal fistula**				
Early	1 (0,7)	0 (0,0)	1 (1,6)	0,45
Late	2 (1,4)	0 (0,0)	2 (3,1)	0,2
**Genitourinary**,** hydronephrosis**				
Early	1 (0,7)	0 (0,0)	1 (1,6)	0,45
Late	1 (0,7)	1 (1,3)	0 (0,0)	1,00
**Genitourinary**,** ureteroileal anastomosis leakage**				
Early	1 (0,7)	0 (0,0)	1 (1,6)	0,45
Late	-	-	-	-
**Genitourinary**,** ureteral stricture**				
Early	2 (1,4)	1 (1,3)	1 (1,6)	1,00
Late	4 (2,7)	3 (3,9)	1 (1,6)	0,63
**Genitourinary**,** entero-urethral anastomosis leakage**				
Early	4 (2,7)	1 (1,3)	3 (4,7)	0,33
Late	-	-	-	-
**Genitourinary**,** stones in the neobladder**				
Early	-	-	-	-
Late	1 (0,7)	1 (1,3)	0 (0,0)	1,00
**Genitourinary**,** urethrovaginal fistula**				
Early	-	-	-	-
Late	1 (0,7)	0 (0,0)	1 (1,6)	0,45
**Pulmonary**,** apnea**				
Early	1 (0,7)	1 (1,3)	0 (0,0)	1,00
Late	-	-	-	-
**Vascular**,** bleeding**				
Early	1 (0,7)	0 (0,0)	1 (1,6)	0,45
Late	-	-	-	-
**Infection**,** urinary tract infection**				
Early	-	-	-	-
Late	1 (0,7)	0 (0,0)	1 (1,6)	0,45

## Discussion

In this large, multi-institutional study, we investigated the outcomes of female patients who underwent RARC and intracorporeal orthotopic neobladder reconstruction. We found that this surgical approach resulted in reduced incontinence rates as compared with non-organ-preserving technique. In addition, organ-preserving technique showed comparable short-term oncological and complication rates compared to previously reported data on patients operated with incontinent urinary diversion [7, 12].

Historically, the use of orthotopic neobladder in females has been less common than in males, mainly due to concerns on the possible increased risk of local recurrences and postoperative voiding dysfunction [13]. Indeed, Gore et al. demonstrated with nearly 4000 patients from the SEER database who had undergone radical cystectomy that males have higher likelihood to undergo continent reconstruction than women (OR 1,31) [8]. However, even if cross comparison between studies cannot be made, continence rates in our study are close to the rates presented by Martini et al. among men operated with RARC and orthotopic neobladder reconstruction [14], however, in our study, the hypercontinence rates in females were higher. Interestingly, pelvic organ-preserving procedure led to reduced incontinence rates (15% vs. 35%) but also to higher hypercontinence rate compared to non-organ-preserving procedure. In our study, the hypercontinence rate for POP was 28% during daytime and 25% during nighttime as compared with 15% and 9% for non-POP, respectively. These results are consistent with findings from previous studies, which have demonstrated good functional outcomes [10, 15–17] after POP. Most of these studies report patients operated with open approach.

The adoption of pelvic organ-preserving RARC has been slow, mainly due to apprehensions about positive surgical margins and potential secondary malignancies in gynecological reproductive organs. In earlier studies, positive surgical margins have occurred in female cystectomies in around 4–6% of the cases [18, 19], which aligns with our study, wherein we demonstrate a comparable incidence of positive surgical margins in both the pelvic organ-preserving and non-organ-preserving groups (5% in each group). Since our results are based on retrospective data, there is certainly a selection-bias to be considered when making a direct comparison between these groups. Although POP was performed in our study more often to patients with NMIBC, nearly half of the patients had muscle-invasive cancer, which suggests that POP could be safely performed also in selected patients with muscle-invasive tumors when the anatomical location of the tumor is suitable. The lack of long-term oncological follow-up data in our study, however, impedes more profound analysis. In a recent propensity-score matched analysis, POP and non-POP showed similar oncological survival [20], and the risk for gynecological malignancies has not been shown to increase in patients operated with RC compared to control population [21–23].

The impact of radical cystectomy on sexual function and health-related quality of life (HRQoL) in women is underreported. Studies in men with orthotopic neobladder reconstruction have shown improvements in HRQoL compared to ileal conduit diversion, with similar morbidity and mortality profiles [7, 12, 24]. Sexual dysfunction has been a concern for patients with neobladder reconstruction [25], but pelvic organ-preserving approaches may help mitigate this issue.

There are several limitations to our study. Firstly, centers included in this study are high-volume centers with highly experienced surgeons with the capability of perform orthotopic neobladder with or without preserving pelvic organs, and thus results may be difficult to reproduce in smaller institutions. Secondly, the retrospective nature of data collection introduces bias, as decisions regarding pelvic organ-preservation were likely influenced by patient characteristics. Thus, direct comparisons of the pelvic organ-preserving and non-organ-preserving groups may not be entirely reliable. Furthermore, the classification of complications retrospectively without a uniform praxis may lead to underreporting of the complications. Lastly, the absence of long term oncological follow up data, and information on metastasis prevents a complete analysis of the oncological outcomes.

Our study demonstrates that RARC with intracorporeal orthotopic neobladder reconstruction in females can achieve good functional, oncological and complication outcomes. Furthermore, POP reduces incontinence but may result in higher rates of hypercontinence. RARC with intracorporeal neobladder could be considered in females more often as than it is done at present. Further studies with longer follow-up and additional oncological data are needed to confirm these findings and provide more comprehensive comparisons between pelvic organ-preserving and non-organ-preserving approaches.

## Electronic supplementary material

Below is the link to the electronic supplementary material.

Supplementary Material 1

Supplementary Material 2

Supplementary Material 3

Supplementary Material 4

## Data Availability

No datasets were generated or analysed during the current study.
