# Supplementary figures and images for: Outcomes after robot-assisted radical cystectomy with orthotopic neobladder in women
Source: World J Urol. 2024 Nov 2;42(1):617. doi: 10.1007/s00345-024-05339-w (PMC11531442; doi:10.1007/s00345-024-05339-w)

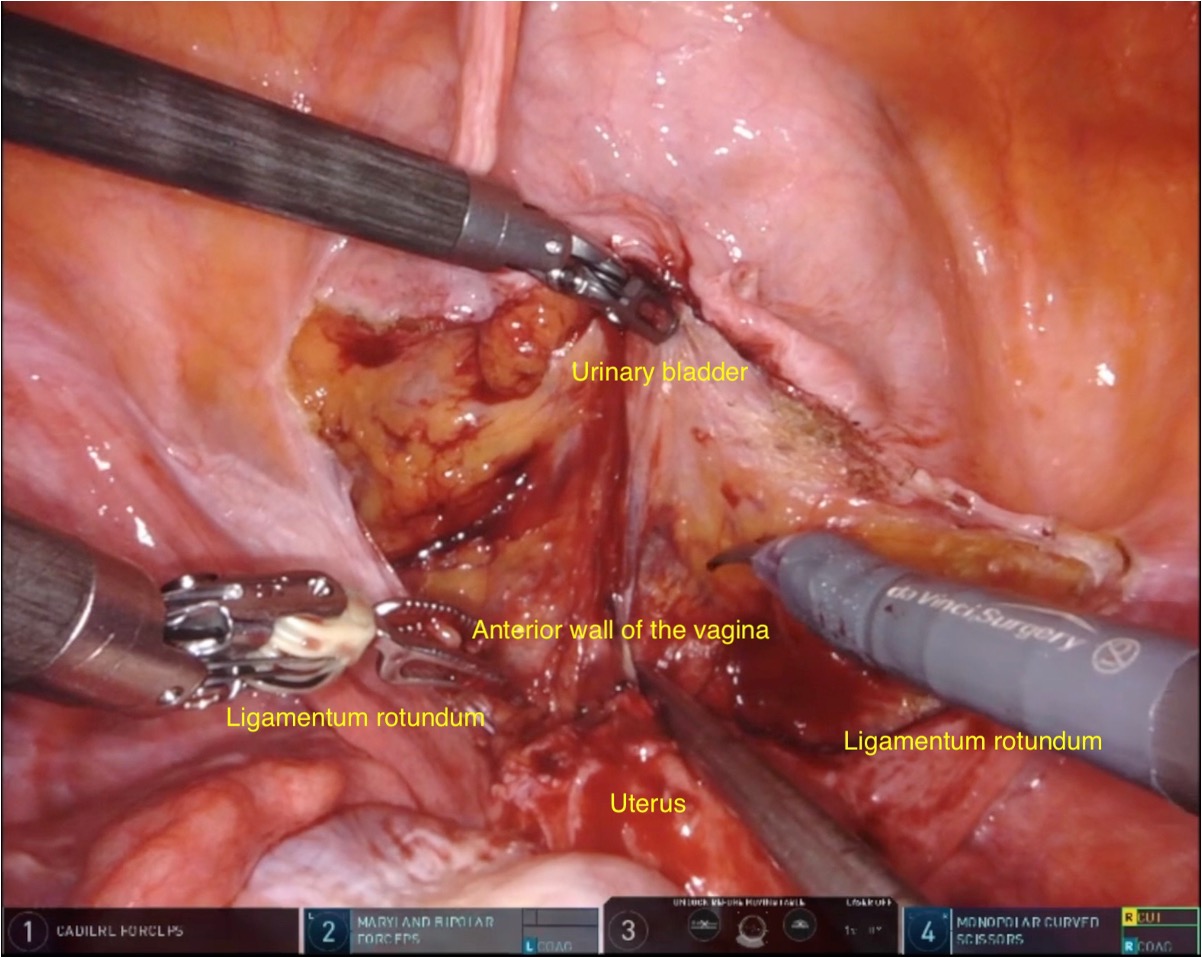

Supplement: Supplementary file 1 — Supplementary Material 1 [file 345_2024_5339_MOESM1_ESM.jpg]

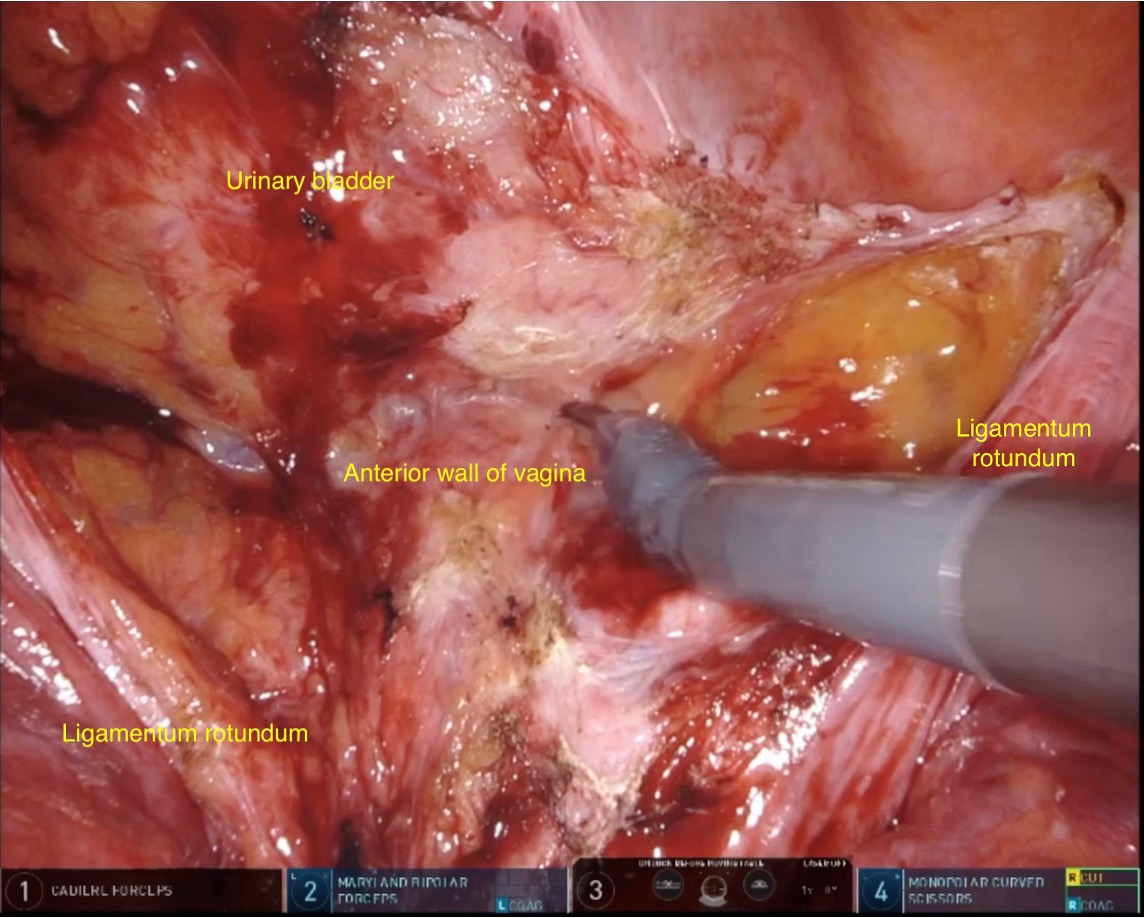

Supplement: Supplementary file 2 — Supplementary Material 2 [file 345_2024_5339_MOESM2_ESM.jpg]

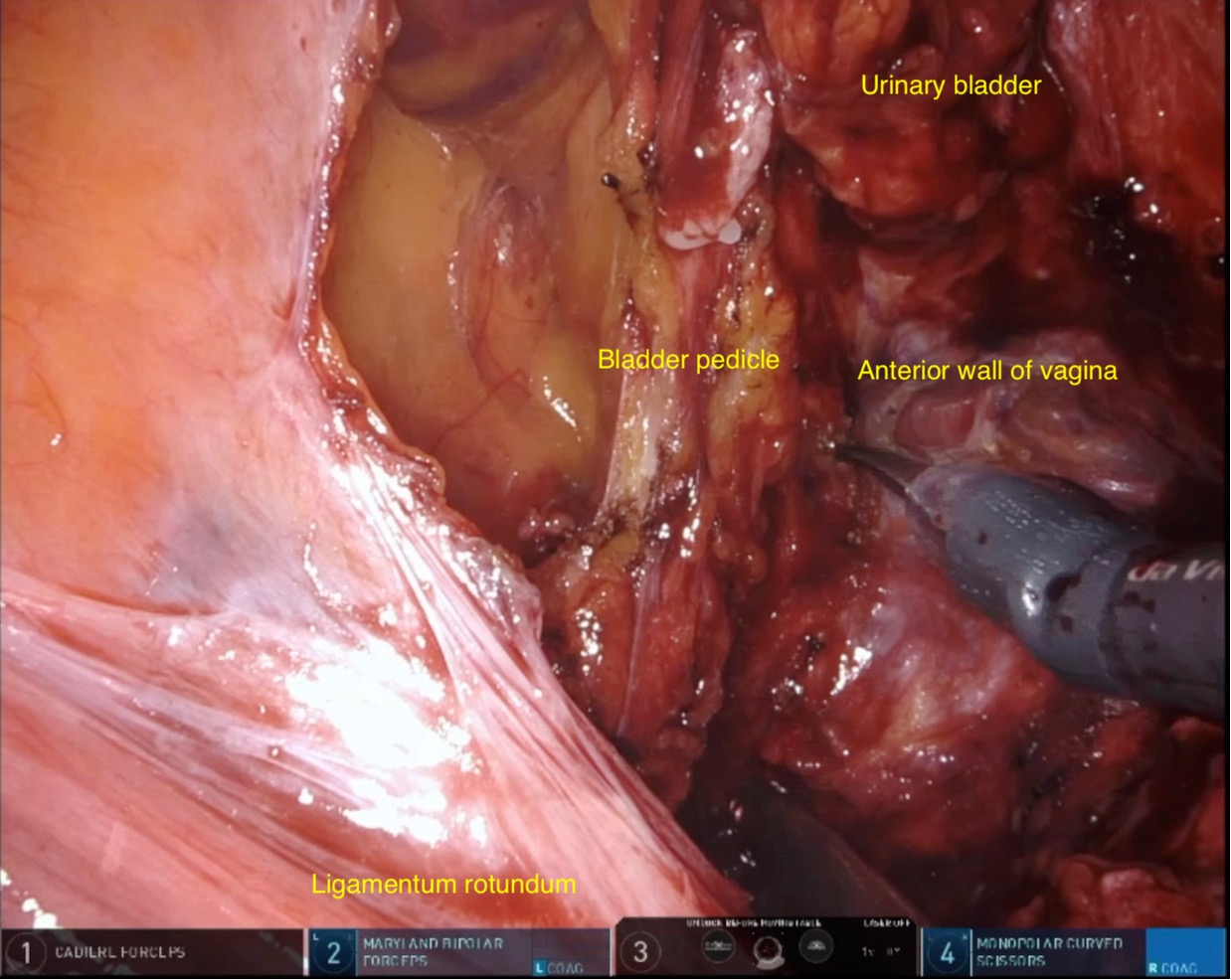

Supplement: Supplementary file 3 — Supplementary Material 3 [file 345_2024_5339_MOESM3_ESM.jpg]

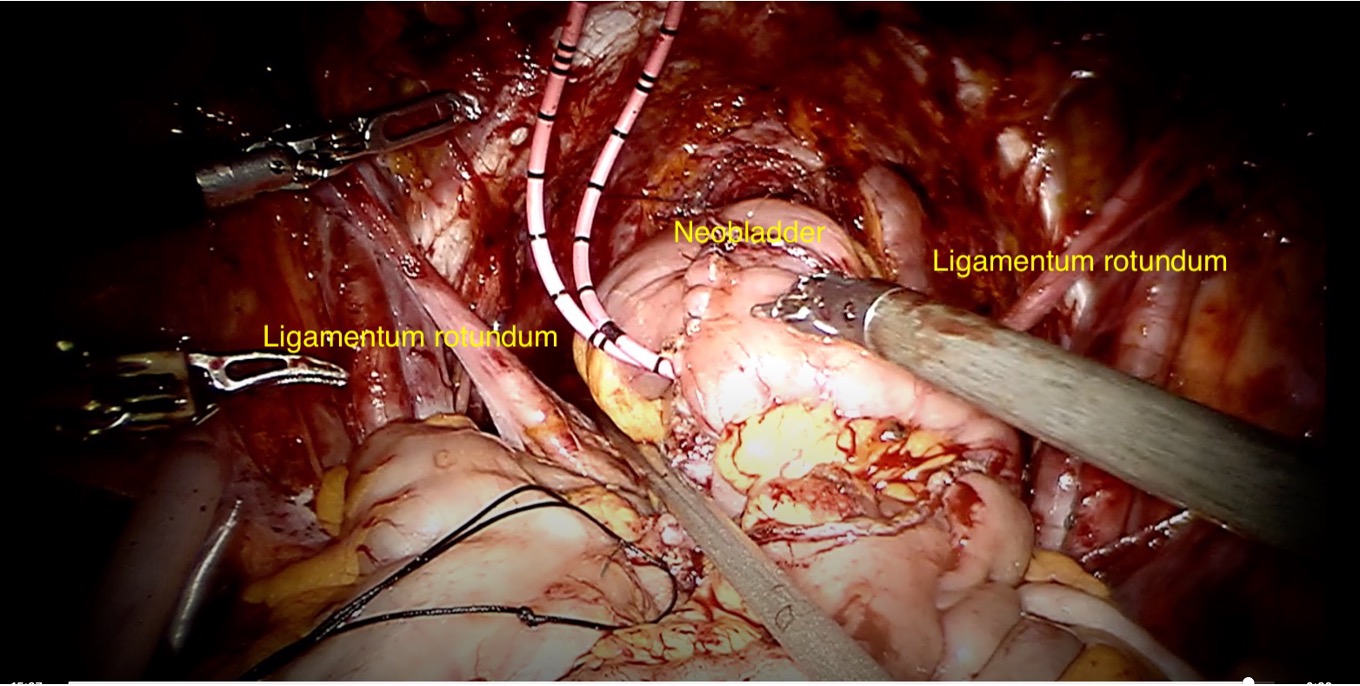

Supplement: Supplementary file 4 — Supplementary Material 4 [file 345_2024_5339_MOESM4_ESM.jpg]
